# Eletrocardiograma como Preditor de Mortalidade em Indivíduos com Hipertensão Pulmonar

**DOI:** 10.36660/abc.20240548

**Published:** 2024-09-17

**Authors:** Frederico José Neves Mancuso

**Affiliations:** 1 Universidade Federal de São Paulo Escola Paulista de Medicina São Paulo SP Brasil Universidade Federal de São Paulo – Escola Paulista de Medicina (UNIFESP/EPM), São Paulo, SP – Brasil

**Keywords:** Eletrocardiografia, Hipertensão Pulmonar

Hipertensão pulmonar (HP) é definida como pressão média de artéria pulmonar maior que 20 mmHg em avaliação invasiva por cateterismo cardíaco direito.^1,2^

Diretrizes sugerem que pacientes com HP devem ser estratificados quanto ao seu prognóstico tanto no momento do diagnóstico, como ao longo do seguimento, com ênfase na avaliação clínica pela classe funcional, teste de caminhada de 6 minutos e níveis de peptídeo natriurético.^2^

Outros parâmetros, obtidos por teste cardiopulmonar, ecocardiografia, ressonância magnética cardíaca ou dados hemodinâmicos invasivos também são recomendados para essa estratificação de risco.^1,3^

Destaca-se que a estratificação de risco, além de determinar o prognóstico dos pacientes, é fundamental para guiar e ajustar a terapêutica durante o seguimento desses pacientes, determinando o uso de monoterapia, terapia dupla ou terapia tripla; conforme ação sobre a via do óxido nítrico, via da prostaciclina e/ou via da endotelina.^1,2^

É interessante notar que o eletrocardiograma (ECG), apesar de ser um exame simples, de muito baixo custo e não invasivo,^4,5^ não faz parte das ferramentas geralmente sugeridas para a estratificação de risco e prognóstico destes pacientes.^2^

Em pacientes com HP, o ECG pode auxiliar tanto na suspeita diagnóstica, mostrando sinais como onda P pulmonale, desvio do eixo do QRS para a direita, sobrecarga de ventrículo direito, bloqueio de ramo direito (BRD), alteração da repolarização em parede inferior ou V1-V4,^2^ como na avaliação prognóstica.^2^

Assim, é importante a publicação de Koyun et al. que estudou o valor prognóstico do ECG em pacientes com HP. No estudo, os autores avaliaram especificamente o intervalo de tempo RS.^6^ O intervalo RS é calculado entre o início do QRS até o ponto mais inferior da onda S ou S´, sendo considerado aquele maior entre as derivações D1, D2, D3, AVL, AVF, V4, V5 e V6.^6^

Park et al. avaliaram 364 pacientes com BRD que realizaram ecocardiograma, excluindo pacientes com disfunção ventricular esquerda, demonstrando que a duração do QRS se correlacionada com disfunção do ventrículo direito,^7^ um fator conhecido para desfecho desfavorável na HP.^2,4^

Em outro estudo eletrocardiográfico em pacientes com HP (idiopática ou por doença do tecido conjuntivo), Waligóra et al. demonstraram que a maior duração do QRS se associa a maior mortalidade nestes pacientes (p = 0,01).^8^

Especificamente, o intervalo RS no ECG foi avaliado em 216 pacientes com tromboembolismo pulmonar (TEP), junto como outros parâmetros, como idade, presença de neoplasia, escore PESI, fração de ejeção do ventrículo esquerdo, uso de terapia trombolítica, entre outros. Na análise multivariada, a duração do intervalo RS na admissão foi preditor independente de mortalidade em um mês (p = 0,02).^9^

Estimulados pelos achados na HP aguda do TEP, Koyun et al. avaliaram o valor prognóstico da duração do intervalo RS na HP em artigo publicado neste número.^6^ Foram incluídos 143 pacientes com hipertensão arterial pulmonar (grupo 1) e 143 indivíduos saudáveis (grupo controle). Em análise multivariada, a duração do intervalo RS se mostrou um preditor independente de mortalidade nestes pacientes (p < 0,001), após seguimento médio de 30,4 meses.^6^ O intervalo RS teve correlação significativa com a dilatação do ventrículo direito e a pressão sistólica em artéria pulmonar.

Estes achados são importantes, já que trazem a possibilidade do uso de uma ferramenta de baixo custo e amplamente disponível para auxiliar na determinação prognóstico de pacientes com HP. Deve-se considerar que este é um estudo retrospectivo, e, portanto, estes achados devem ser validados em estudo prospectivo antes de ser utilizado na prática clínica. Também é importante que seja avaliado como o ECG pode ajudar na escolha da terapia destes pacientes.
