# Supplementary material for: Eletrocardiograma como Preditor de Mortalidade em Indivíduos com Hipertensão Pulmonar
Source: Arq Bras Cardiol. 2024 Sep 17;121(9):e20240548. [Article in Portuguese] doi: 10.36660/abc.20240548 (PMC11495588; doi:10.36660/abc.20240548)
Supplement: Translation

# Translation: Electrocardiogram as a Predictor of Mortality in Individuals with Pulmonary Hypertension

**DOI:** 10.36660/abc.20240548i

**Authors:** Frederico José Neves Mancuso

**Affiliations:** 1 São Paulo SP Brazil Universidade Federal de São Paulo – Escola Paulista de Medicina (UNIFESP/EPM), São Paulo, SP – Brazil

**Keywords:** Electrocardiography, Hypertension, Pulmonary

Pulmonary hypertension (PH) is defined as mean pulmonary artery pressure greater than 20 mmHg in invasive assessment by right heart catheterization.^1,2^

Guidelines suggest that patients with PH should be stratified according to their prognosis both at the time of diagnosis and throughout follow-up, with emphasis on clinical assessment by functional class, 6-minute walk test, and natriuretic peptide levels.^2^

Other parameters, obtained by cardiopulmonary testing, echocardiography, cardiac magnetic resonance, or invasive hemodynamic data, are also recommended for this risk stratification.^1,3^

It is noteworthy that risk stratification, in addition to determining the patient's prognosis, is essential to guide and adjust therapy during the follow-up of these patients, determining the use of monotherapy, double therapy, or triple therapy; according to action on the nitric oxide pathway, prostacyclin pathway and/or endothelin pathway.^1,2^

It is interesting to note that the electrocardiogram (ECG), despite being a simple, very low-cost, and non-invasive test,^4,5^ is not part of the tools generally suggested for risk stratification and prognosis of these patients.^2^

In patients with PH, the ECG can help with diagnostic suspicion, showing signs such as P pulmonale wave, deviation of the QRS axis to the right, right ventricular overload, right bundle branch block (RBBB), change in repolarization in the inferior wall or V1-V4,^2^ as in prognostic assessment.^2^

Therefore, the publication by Koyun et al., who studied the prognostic value of ECG in patients with PH, is important. In the study, the authors specifically evaluated the RS^6^ time frame. The RS interval is calculated between the beginning of the QRS to the lowest point of the S or S’ wave, being considered the longest between leads D1, D2, D3, AVL, AVF, V4, V5, and V6.^6^

Park et al. evaluated 364 patients with RBBB who underwent echocardiography, excluding patients with left ventricular dysfunction, demonstrating that QRS duration is correlated with right ventricular dysfunction,^7^ a known factor for unfavorable outcome in PH.^2,4^

In another electrocardiographic study in patients with PH (idiopathic or due to connective tissue disease), Waligora et al. demonstrated that longer QRS duration is associated with higher mortality in these patients (p = 0.01).^8^

Specifically, the RS interval on the ECG was evaluated in 216 patients with pulmonary thromboembolism (PTE), along with other parameters, such as age, presence of neoplasia, PESI score, left ventricular ejection fraction, use of thrombolytic therapy, among others. In multivariate analysis, the duration of the RS interval at admission was an independent predictor of one-month mortality (p = 0.02).^9^

Spurred by findings in acute PH from PTE, Koyun et al. evaluated the prognostic value of the duration of the RS interval in PH in an article published in this issue.^6^ 143 patients with pulmonary arterial hypertension (group 1) and 143 healthy individuals (control group) were included. In multivariate analysis, the duration of the RS interval proved to be an independent predictor of mortality in these patients (p < 0.001), after a mean follow-up of 30.4 months.^6^ The RS interval had a significant correlation with right ventricular dilation and systolic pressure in the pulmonary artery.

These findings are important, as they bring the possibility of using a low-cost and widely available tool to help determine the prognosis of patients with PH. It must be considered that this is a retrospective study, and therefore, these findings must be validated in a prospective study before being used in clinical practice. It is also important to evaluate how the ECG can help in choosing therapy for these patients.
